# 2,6-Bis(3-methoxy­phen­yl)-3-methyl­piperidin-4-one

**DOI:** 10.1107/S1600536809045346

**Published:** 2009-11-04

**Authors:** P. Nithya, F. Nawaz Khan, Motakatla Novanna, Venkatesha R. Hathwar, Seik Weng Ng

**Affiliations:** a Chemistry Division, School of Science and Humanities, VIT University, Vellore 632 014, Tamil Nadu, India; b Solid State and Structural Chemistry Unit, Indian Institute of Science, Bangalore 560 012, Karnataka, India; c Department of Chemistry, University of Malaya, 50603 Kuala Lumpur, Malaysia

## Abstract

In the mol­ecule of the title compound, C_20_H_23_NO_3_, the bulky methoxy­phenyl substituents at the equatorial 2,6-positions crowd the vicinity of the equatorial amino H atom and prevent it from forming inter­molecular hydrogen bonds. The piperidine ring adopts a distorted chair conformation.

## Related literature

For the crystal structure of a related piperidinone compound, see: Nithya *et al.* (2009 ▶).
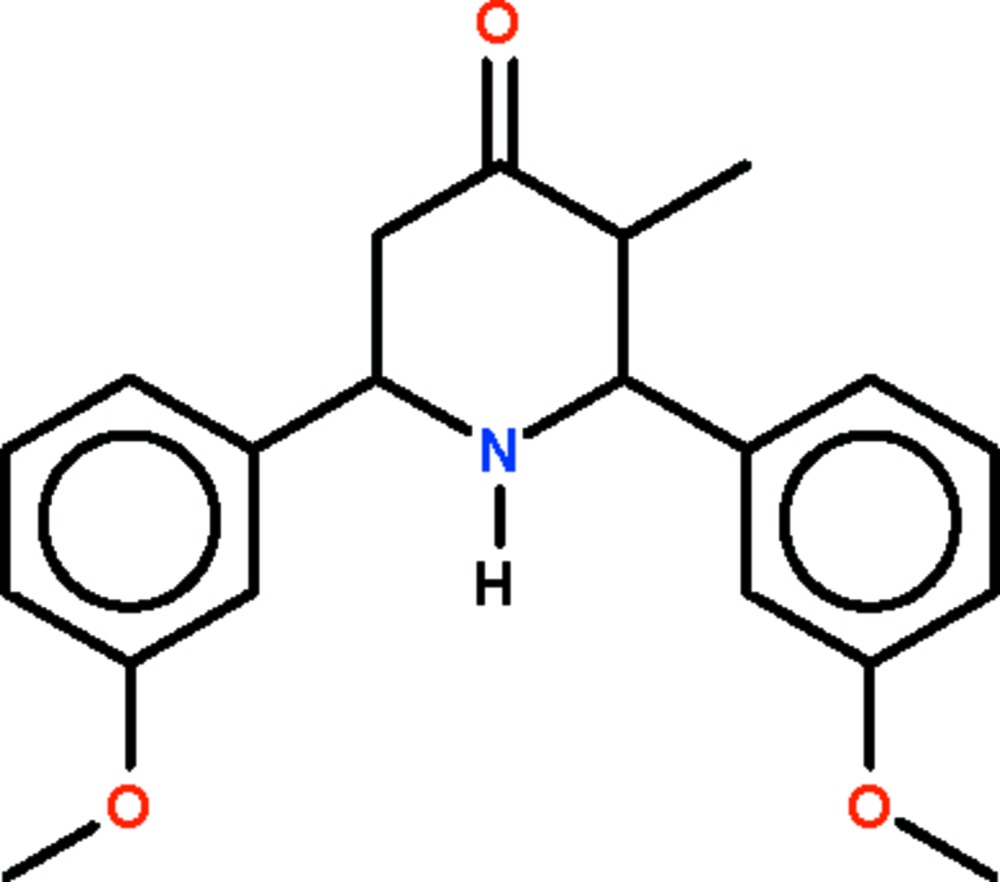


## Experimental

### 

#### Crystal data

C_20_H_23_NO_3_
*M* *_r_* = 325.39
Monoclinic, 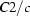

*a* = 28.695 (3) Å
*b* = 10.9717 (12) Å
*c* = 11.3946 (13) Å
β = 95.078 (2)°
*V* = 3573.3 (7) Å^3^
*Z* = 8
Mo *K*α radiation
μ = 0.08 mm^−1^
*T* = 290 K
0.35 × 0.12 × 0.08 mm

#### Data collection

Bruker SMART CCD area-detector diffractometer
Absorption correction: none
12581 measured reflections
3148 independent reflections
1751 reflections with *I* > 2σ(*I*)
*R*_int_ = 0.058

#### Refinement

*R*[*F* ^2^ > 2σ(*F* ^2^)] = 0.063
*wR*(*F* ^2^) = 0.154
*S* = 1.04
3148 reflections
224 parameters
H atoms treated by a mixture of independent and constrained refinement
Δρ_max_ = 0.18 e Å^−3^
Δρ_min_ = −0.14 e Å^−3^

### 

Data collection: *SMART* (Bruker, 2004 ▶); cell refinement: *SAINT* (Bruker, 2004 ▶); data reduction: *SAINT*; program(s) used to solve structure: *SHELXS97* (Sheldrick, 2008 ▶); program(s) used to refine structure: *SHELXL97* (Sheldrick, 2008 ▶); molecular graphics: *X-SEED* (Barbour, 2001 ▶); software used to prepare material for publication: *publCIF* (Westrip, 2009 ▶).

## Supplementary Material

Crystal structure: contains datablocks global, I. DOI: 10.1107/S1600536809045346/ci2955sup1.cif

Structure factors: contains datablocks I. DOI: 10.1107/S1600536809045346/ci2955Isup2.hkl

Additional supplementary materials:  crystallographic information; 3D view; checkCIF report

## Figures and Tables

**Fig. 1.**
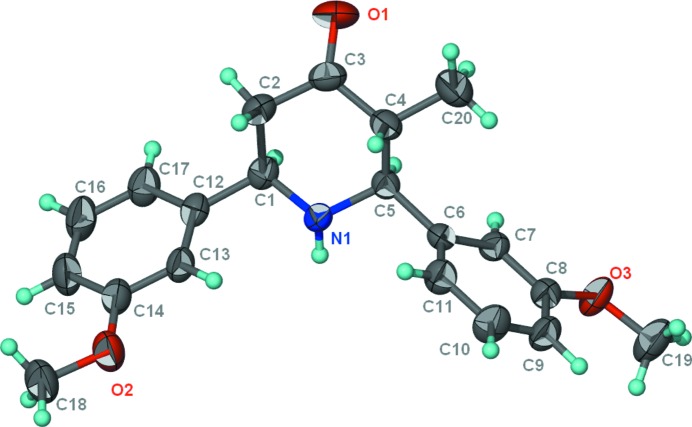
Displacement ellipsoid plot (Barbour, 2001) of C20H23NO3 at the 50% probability level. H atoms are drawn as spheres of arbitrary radius.

## supplementary crystallographic information

### Experimental

Ammonium acetate (1 mmol), *m*-methoxybenzaldehyde (2 mmol) and ethylmethyl ketone (1 mmol) was heated until the colour of the solution turned yellow. After the completion of the reaction (as monitored by TLC), the product was dissolved in ether (10 ml). The solution was treated with aqueous hydrochloric acid [20 ml, 1:1 (*v*/*v*)]. The hydrochloride salt of the piperidin-4-one was collected and washed with ether. The base was liberated from an alcohol solution of the hydrochloride by the addition of a slight excess of aqueous ammonia at 273 K. The product was collected and recrystallized from ethanol.

### Refinement

C-bound H-atoms were placed in calculated positions (C-H = 0.93–0.98 Å) and were included in the refinement in the riding model approximation, with *U_iso_*(H) set to 1.2–1.5*U_eq_*(C).

### Crystal data

**Table:**

C_20_H_23_NO_3_	*F*(000) = 1392
*M**_r_* = 325.39	*D*_x_ = 1.210 Mg m^−^^3^
Monoclinic, *C*2/*c*	Mo *K*α radiation, λ = 0.71073 Å
Hall symbol: -C 2yc	Cell parameters from 1065 reflections
*a* = 28.695 (3) Å	θ = 2.0–20.8°
*b* = 10.9717 (12) Å	µ = 0.08 mm^−^^1^
*c* = 11.3946 (13) Å	*T* = 290 K
β = 95.078 (2)°	Plate, colourless
*V* = 3573.3 (7) Å^3^	0.35 × 0.12 × 0.08 mm
*Z* = 8	

### Data collection

**Table:**

Bruker SMART CCD area-detector diffractometer	1751 reflections with *I* > 2σ(*I*)
Radiation source: fine-focus sealed tube	*R*_int_ = 0.058
graphite	θ_max_ = 25.0°, θ_min_ = 1.4°
φ and ω scans	*h* = −33→34
12581 measured reflections	*k* = −13→11
3148 independent reflections	*l* = −13→13

### Refinement

**Table:**

Refinement on *F*^2^	Primary atom site location: structure-invariant direct methods
Least-squares matrix: full	Secondary atom site location: difference Fourier map
*R*[*F*^2^ > 2σ(*F*^2^)] = 0.063	Hydrogen site location: inferred from neighbouring sites
*wR*(*F*^2^) = 0.154	H atoms treated by a mixture of independent and constrained refinement
*S* = 1.04	*w* = 1/[σ^2^(*F*_o_^2^) + (0.0658*P*)^2^ + 0.6442*P*] where *P* = (*F*_o_^2^ + 2*F*_c_^2^)/3
3148 reflections	(Δ/σ)_max_ = 0.001
224 parameters	Δρ_max_ = 0.18 e Å^−^^3^
0 restraints	Δρ_min_ = −0.14 e Å^−^^3^

### Fractional atomic coordinates and isotropic or equivalent isotropic displacement parameters (Å^2^)

**Table:**

	*x*	*y*	*z*	*U*_iso_*/*U*_eq_	
O1	0.58419 (9)	0.8518 (2)	0.9145 (2)	0.1002 (10)	
O2	0.76371 (8)	0.5426 (2)	0.51929 (18)	0.0750 (7)	
O3	0.57121 (8)	0.17943 (18)	1.10361 (17)	0.0647 (6)	
N1	0.65515 (8)	0.5548 (2)	0.83450 (19)	0.0438 (6)	
H1N	0.6737 (9)	0.493 (2)	0.850 (2)	0.042 (8)*	
C1	0.68070 (10)	0.6693 (3)	0.8526 (2)	0.0460 (7)	
H1	0.6910	0.6772	0.9366	0.055*	
C2	0.64767 (11)	0.7743 (3)	0.8172 (3)	0.0585 (9)	
H2A	0.6627	0.8505	0.8419	0.070*	
H2B	0.6420	0.7760	0.7320	0.070*	
C3	0.60185 (12)	0.7651 (3)	0.8696 (3)	0.0588 (9)	
C4	0.57864 (10)	0.6416 (3)	0.8627 (2)	0.0488 (8)	
H4	0.5697	0.6251	0.7792	0.059*	
C5	0.61533 (9)	0.5452 (2)	0.9056 (2)	0.0405 (7)	
H5	0.6262	0.5630	0.9878	0.049*	
C6	0.59593 (9)	0.4172 (2)	0.8989 (2)	0.0392 (7)	
C7	0.59208 (9)	0.3507 (2)	1.0002 (2)	0.0429 (7)	
H7	0.6019	0.3850	1.0729	0.051*	
C8	0.57388 (10)	0.2338 (3)	0.9957 (2)	0.0456 (7)	
C9	0.56020 (11)	0.1801 (3)	0.8898 (3)	0.0573 (8)	
H9	0.5487	0.1009	0.8864	0.069*	
C10	0.56400 (11)	0.2471 (3)	0.7879 (3)	0.0646 (9)	
H10	0.5544	0.2123	0.7153	0.078*	
C11	0.58152 (10)	0.3632 (3)	0.7912 (2)	0.0525 (8)	
H11	0.5838	0.4061	0.7216	0.063*	
C12	0.72330 (10)	0.6733 (3)	0.7837 (2)	0.0445 (7)	
C13	0.72600 (10)	0.6065 (3)	0.6817 (2)	0.0500 (8)	
H13	0.7013	0.5558	0.6550	0.060*	
C14	0.76509 (11)	0.6143 (3)	0.6189 (3)	0.0549 (8)	
C15	0.80184 (12)	0.6882 (3)	0.6568 (3)	0.0686 (10)	
H15	0.8284	0.6922	0.6155	0.082*	
C16	0.79871 (12)	0.7568 (3)	0.7577 (3)	0.0716 (10)	
H16	0.8232	0.8085	0.7834	0.086*	
C17	0.76009 (11)	0.7498 (3)	0.8206 (3)	0.0597 (9)	
H17	0.7587	0.7967	0.8882	0.072*	
C18	0.80099 (13)	0.5566 (3)	0.4451 (3)	0.0848 (12)	
H18A	0.8021	0.6396	0.4189	0.127*	
H18B	0.7958	0.5036	0.3782	0.127*	
H18C	0.8301	0.5359	0.4885	0.127*	
C19	0.55104 (15)	0.0625 (3)	1.1084 (3)	0.0877 (12)	
H19A	0.5192	0.0654	1.0746	0.132*	
H19B	0.5518	0.0365	1.1890	0.132*	
H19C	0.5684	0.0060	1.0650	0.132*	
C20	0.53454 (12)	0.6374 (3)	0.9252 (3)	0.0826 (11)	
H20A	0.5421	0.6511	1.0079	0.124*	
H20B	0.5201	0.5589	0.9136	0.124*	
H20C	0.5134	0.6994	0.8939	0.124*	

### Atomic displacement parameters (Å^2^)

**Table:**

	*U*^11^	*U*^22^	*U*^33^	*U*^12^	*U*^13^	*U*^23^
O1	0.119 (2)	0.0536 (16)	0.135 (2)	0.0140 (15)	0.0511 (19)	−0.0246 (15)
O2	0.0798 (17)	0.0788 (16)	0.0713 (14)	−0.0245 (13)	0.0343 (13)	−0.0180 (13)
O3	0.0959 (17)	0.0445 (13)	0.0538 (13)	−0.0223 (12)	0.0074 (11)	0.0049 (10)
N1	0.0422 (15)	0.0342 (15)	0.0560 (15)	0.0009 (13)	0.0100 (12)	0.0041 (12)
C1	0.0515 (19)	0.0455 (18)	0.0403 (15)	−0.0075 (16)	0.0001 (14)	−0.0005 (13)
C2	0.069 (2)	0.0394 (19)	0.068 (2)	−0.0052 (16)	0.0137 (17)	−0.0052 (15)
C3	0.071 (2)	0.042 (2)	0.064 (2)	0.0102 (18)	0.0113 (17)	−0.0019 (15)
C4	0.0485 (19)	0.051 (2)	0.0485 (17)	0.0065 (16)	0.0123 (14)	−0.0005 (14)
C5	0.0421 (17)	0.0418 (17)	0.0381 (14)	−0.0016 (14)	0.0074 (12)	−0.0017 (12)
C6	0.0373 (16)	0.0382 (17)	0.0431 (16)	0.0028 (13)	0.0092 (13)	−0.0022 (13)
C7	0.0485 (18)	0.0393 (18)	0.0410 (16)	−0.0024 (14)	0.0049 (13)	−0.0036 (13)
C8	0.0482 (19)	0.0446 (19)	0.0447 (16)	−0.0016 (15)	0.0075 (13)	−0.0012 (14)
C9	0.068 (2)	0.0440 (19)	0.060 (2)	−0.0164 (16)	0.0096 (16)	−0.0051 (15)
C10	0.086 (3)	0.060 (2)	0.0469 (18)	−0.019 (2)	0.0026 (16)	−0.0138 (16)
C11	0.063 (2)	0.055 (2)	0.0407 (17)	−0.0082 (17)	0.0069 (14)	−0.0025 (14)
C12	0.0453 (18)	0.0438 (17)	0.0438 (16)	−0.0059 (15)	0.0006 (14)	0.0074 (13)
C13	0.0480 (19)	0.0487 (19)	0.0536 (18)	−0.0162 (15)	0.0067 (15)	−0.0026 (14)
C14	0.057 (2)	0.053 (2)	0.0560 (19)	−0.0118 (17)	0.0130 (16)	0.0019 (16)
C15	0.060 (2)	0.077 (2)	0.072 (2)	−0.019 (2)	0.0216 (18)	0.0035 (19)
C16	0.062 (2)	0.077 (2)	0.077 (2)	−0.032 (2)	0.0088 (19)	−0.002 (2)
C17	0.061 (2)	0.064 (2)	0.0535 (18)	−0.0187 (19)	0.0034 (16)	−0.0029 (16)
C18	0.089 (3)	0.094 (3)	0.077 (2)	−0.020 (2)	0.042 (2)	−0.012 (2)
C19	0.140 (4)	0.054 (2)	0.071 (2)	−0.031 (2)	0.021 (2)	0.0032 (18)
C20	0.074 (3)	0.076 (3)	0.103 (3)	0.017 (2)	0.039 (2)	0.011 (2)

### Geometric parameters (Å, °)

**Table:**

O1—C3	1.212 (3)	C9—C10	1.388 (4)
O2—C14	1.379 (3)	C9—H9	0.93
O2—C18	1.429 (3)	C10—C11	1.368 (4)
O3—C8	1.375 (3)	C10—H10	0.93
O3—C19	1.411 (3)	C11—H11	0.93
N1—C1	1.460 (3)	C12—C13	1.382 (4)
N1—C5	1.462 (3)	C12—C17	1.384 (4)
N1—H1N	0.87 (3)	C13—C14	1.385 (4)
C1—C12	1.511 (4)	C13—H13	0.93
C1—C2	1.524 (4)	C14—C15	1.369 (4)
C1—H1	0.98	C15—C16	1.384 (4)
C2—C3	1.495 (4)	C15—H15	0.93
C2—H2A	0.97	C16—C17	1.374 (4)
C2—H2B	0.97	C16—H16	0.93
C3—C4	1.509 (4)	C17—H17	0.93
C4—C20	1.507 (4)	C18—H18A	0.96
C4—C5	1.541 (4)	C18—H18B	0.96
C4—H4	0.98	C18—H18C	0.96
C5—C6	1.511 (3)	C19—H19A	0.96
C5—H5	0.98	C19—H19B	0.96
C6—C7	1.379 (3)	C19—H19C	0.96
C6—C11	1.392 (3)	C20—H20A	0.96
C7—C8	1.384 (4)	C20—H20B	0.96
C7—H7	0.93	C20—H20C	0.96
C8—C9	1.368 (4)		
			
C14—O2—C18	117.2 (2)	C10—C9—H9	120.9
C8—O3—C19	119.0 (2)	C11—C10—C9	121.7 (3)
C1—N1—C5	113.0 (2)	C11—C10—H10	119.2
C1—N1—H1N	110.2 (17)	C9—C10—H10	119.2
C5—N1—H1N	109.0 (16)	C10—C11—C6	120.0 (3)
N1—C1—C12	111.8 (2)	C10—C11—H11	120.0
N1—C1—C2	108.7 (2)	C6—C11—H11	120.0
C12—C1—C2	110.7 (2)	C13—C12—C17	118.6 (3)
N1—C1—H1	108.5	C13—C12—C1	122.0 (3)
C12—C1—H1	108.5	C17—C12—C1	119.4 (3)
C2—C1—H1	108.5	C12—C13—C14	120.6 (3)
C3—C2—C1	113.0 (2)	C12—C13—H13	119.7
C3—C2—H2A	109.0	C14—C13—H13	119.7
C1—C2—H2A	109.0	C15—C14—O2	124.2 (3)
C3—C2—H2B	109.0	C15—C14—C13	120.7 (3)
C1—C2—H2B	109.0	O2—C14—C13	115.1 (3)
H2A—C2—H2B	107.8	C14—C15—C16	118.6 (3)
O1—C3—C2	122.1 (3)	C14—C15—H15	120.7
O1—C3—C4	121.8 (3)	C16—C15—H15	120.7
C2—C3—C4	116.1 (3)	C17—C16—C15	121.2 (3)
C20—C4—C3	112.9 (3)	C17—C16—H16	119.4
C20—C4—C5	114.0 (2)	C15—C16—H16	119.4
C3—C4—C5	108.3 (2)	C16—C17—C12	120.3 (3)
C20—C4—H4	107.1	C16—C17—H17	119.9
C3—C4—H4	107.1	C12—C17—H17	119.9
C5—C4—H4	107.1	O2—C18—H18A	109.5
N1—C5—C6	110.0 (2)	O2—C18—H18B	109.5
N1—C5—C4	108.7 (2)	H18A—C18—H18B	109.5
C6—C5—C4	112.6 (2)	O2—C18—H18C	109.5
N1—C5—H5	108.5	H18A—C18—H18C	109.5
C6—C5—H5	108.5	H18B—C18—H18C	109.5
C4—C5—H5	108.5	O3—C19—H19A	109.5
C7—C6—C11	118.3 (3)	O3—C19—H19B	109.5
C7—C6—C5	120.4 (2)	H19A—C19—H19B	109.5
C11—C6—C5	121.3 (2)	O3—C19—H19C	109.5
C6—C7—C8	121.1 (3)	H19A—C19—H19C	109.5
C6—C7—H7	119.4	H19B—C19—H19C	109.5
C8—C7—H7	119.4	C4—C20—H20A	109.5
C9—C8—O3	124.5 (3)	C4—C20—H20B	109.5
C9—C8—C7	120.7 (3)	H20A—C20—H20B	109.5
O3—C8—C7	114.8 (2)	C4—C20—H20C	109.5
C8—C9—C10	118.2 (3)	H20A—C20—H20C	109.5
C8—C9—H9	120.9	H20B—C20—H20C	109.5
			
C5—N1—C1—C12	176.5 (2)	C6—C7—C8—C9	1.5 (4)
C5—N1—C1—C2	−61.0 (3)	C6—C7—C8—O3	−178.7 (2)
N1—C1—C2—C3	48.7 (3)	O3—C8—C9—C10	178.6 (3)
C12—C1—C2—C3	171.8 (2)	C7—C8—C9—C10	−1.6 (4)
C1—C2—C3—O1	134.6 (3)	C8—C9—C10—C11	1.0 (5)
C1—C2—C3—C4	−45.8 (4)	C9—C10—C11—C6	−0.2 (5)
O1—C3—C4—C20	−4.6 (4)	C7—C6—C11—C10	0.1 (4)
C2—C3—C4—C20	175.7 (3)	C5—C6—C11—C10	−179.9 (3)
O1—C3—C4—C5	−131.9 (3)	N1—C1—C12—C13	26.1 (4)
C2—C3—C4—C5	48.5 (3)	C2—C1—C12—C13	−95.1 (3)
C1—N1—C5—C6	−170.0 (2)	N1—C1—C12—C17	−156.6 (3)
C1—N1—C5—C4	66.3 (3)	C2—C1—C12—C17	82.1 (3)
C20—C4—C5—N1	177.2 (3)	C17—C12—C13—C14	1.2 (4)
C3—C4—C5—N1	−56.3 (3)	C1—C12—C13—C14	178.5 (3)
C20—C4—C5—C6	55.1 (3)	C18—O2—C14—C15	−6.7 (5)
C3—C4—C5—C6	−178.3 (2)	C18—O2—C14—C13	173.8 (3)
N1—C5—C6—C7	124.1 (3)	C12—C13—C14—C15	0.1 (5)
C4—C5—C6—C7	−114.6 (3)	C12—C13—C14—O2	179.5 (3)
N1—C5—C6—C11	−56.0 (3)	O2—C14—C15—C16	179.3 (3)
C4—C5—C6—C11	65.4 (3)	C13—C14—C15—C16	−1.3 (5)
C11—C6—C7—C8	−0.7 (4)	C14—C15—C16—C17	1.2 (5)
C5—C6—C7—C8	179.2 (2)	C15—C16—C17—C12	0.0 (5)
C19—O3—C8—C9	−3.2 (4)	C13—C12—C17—C16	−1.2 (4)
C19—O3—C8—C7	177.0 (3)	C1—C12—C17—C16	−178.6 (3)

### Hydrogen-bond geometry (Å, °)

**Table:**

*D*—H···*A*	*D*—H	H···*A*	*D*···*A*	*D*—H···*A*
C2—H2B···O3^i^	0.97	2.45	3.170 (4)	131
C19—H19C···O1^ii^	0.96	2.48	3.392 (4)	159

Symmetry codes: (i) *x*, −*y*+1, *z*−1/2; (ii) *x*, *y*−1, *z*.
